# Supplementary material for: Empirical methods for controlling false positives and estimating confidence in ChIP-Seq peaks
Source: BMC Bioinformatics. 2008 Dec 5;9:523. doi: 10.1186/1471-2105-9-523 (PMC2628906; doi:10.1186/1471-2105-9-523)
Supplement: Additional File 1 — A variety of html documents from the USeq web site detailing the available applications, their best usage, output file type descriptions, command line menus, etc. [file 1471-2105-9-523-S1.zip › USeqUserGuides/JavaDoc.zip!/JavaDoc/gata/plotter/ConservedSeqs.html]

ConservedSeqs


|  |  |  |
| --- | --- | --- |
| **Overview** \| **Package** \| **Class** \| **Use** \| **Tree** \| **Deprecated** \| **Index** \| **Help** | |  |
| **PREV CLASS**   **NEXT CLASS** | **FRAMES**    **NO FRAMES**     **All Classes** |  |
| SUMMARY: NESTED \| FIELD \| CONSTR \| METHOD | DETAIL: FIELD \| CONSTR \| METHOD |  |


---


## gata.plotter Class ConservedSeqs

```
java.lang.Object
  gata.plotter.ConservedSeqs
```

---

``` public class ConservedSeqs extends java.lang.Object ```

**Author:**
:   Nix
    Takes an array of Alignments and converts it into two conserved text sequences

---

**Constructor Summary**

`ConservedSeqs(GATAParams params)`


| **Method Summary** | |
| --- | --- |
| `static int[][]` | `convertToBlocks(StartStop[] ss)` |
| `void` | `fetchNewRegions(int leftBaseRef, int rightBaseRef, int leftBaseComp, int rightBaseComp)` |
| `static java.lang.String[]` | `fetchSeq(java.lang.String file)` |
| `boolean` | `fetchSequences()` |
| `void` | `generateConservedSeqs()` |
| `Console` | `getConsole()` |
| `static int[][]` | `growIntArrays(int[][] ints)` |
| `void` | `printSeqsToConsole()` |
| `void` | `printTruncatedSeqsToConsole(int leftBaseRef, int rightBaseRef, int leftBaseComp, int rightBaseComp)` |
| `static java.lang.String` | `reformatSeq(int[][] blocks, java.lang.String seq, java.lang.String format)`             Reformats a sequence given and int[][] containing start stops |

**Methods inherited from class java.lang.Object**

`equals, getClass, hashCode, notify, notifyAll, toString, wait, wait, wait`

**Constructor Detail**

### ConservedSeqs

```
public ConservedSeqs(GATAParams params)
```


**Method Detail**

### fetchSequences

```
public boolean fetchSequences()
```

---


### fetchNewRegions

```
public void fetchNewRegions(int leftBaseRef,
                            int rightBaseRef,
                            int leftBaseComp,
                            int rightBaseComp)
```

---


### generateConservedSeqs

```
public void generateConservedSeqs()
```

---


### printSeqsToConsole

```
public void printSeqsToConsole()
```

---


### printTruncatedSeqsToConsole

```
public void printTruncatedSeqsToConsole(int leftBaseRef,
                                        int rightBaseRef,
                                        int leftBaseComp,
                                        int rightBaseComp)
```

---


### reformatSeq

```
public static java.lang.String reformatSeq(int[][] blocks,
                                           java.lang.String seq,
                                           java.lang.String format)
```

:   Reformats a sequence given and int[][] containing start stops

---


### fetchSeq

```
public static java.lang.String[] fetchSeq(java.lang.String file)
```

---


### convertToBlocks

```
public static int[][] convertToBlocks(StartStop[] ss)
```

---


### growIntArrays

```
public static int[][] growIntArrays(int[][] ints)
```

---


### getConsole

```
public Console getConsole()
```


---


|  |  |  |
| --- | --- | --- |
| **Overview** \| **Package** \| **Class** \| **Use** \| **Tree** \| **Deprecated** \| **Index** \| **Help** | |  |
| **PREV CLASS**   **NEXT CLASS** | **FRAMES**    **NO FRAMES**     **All Classes** |  |
| SUMMARY: NESTED \| FIELD \| CONSTR \| METHOD | DETAIL: FIELD \| CONSTR \| METHOD |  |


---
